# Size-exclusion chromatography–small-angle neutron scattering system optimized for an instrument with medium neutron flux

**DOI:** 10.1107/S1600576725000779

**Published:** 2025-02-11

**Authors:** Ken Morishima, Rintaro Inoue, Tatsuo Nakagawa, Masahiro Shimizu, Ritsuki Sakamoto, Tatsuro Oda, Koichi Mayumi, Masaaki Sugiyama

**Affiliations:** a Institute for Integrated Radiation and Nuclear Science Kyoto University Kumatori, Sennan-gun Osaka590-0494 Japan; b Unisoku Co. Ltd, 2-4-3 Kasugano, Hirakata, Osaka573-0131, Japan; c Graduate School of Science Kyoto University Kitashirakawa, Sakyo-ku Kyoto606-8502 Japan; d The Institute for Solid State Physics University of Tokyo 5-1-5 Kashiwanoha Kashiwa Chiba277-8581 Japan; Tohoku University, Japan

**Keywords:** size-exclusion chromatography–small-angle neutron scattering, SEC–SANS, inverse contrast matching, neutron flux, biomacromolecules

## Abstract

Biomacromolecular solutions inevitably contain impurities in addition to the target biomacromolecules. This has been a major obstacle to achieving high-precision solution scattering measurements. To overcome this problem, small-angle X-ray scattering (SAXS) coupled with size-exclusion chromatography (SEC-SAXS) has been developed. This method involves injecting the solution eluted by SEC directly into a measurement cell and conducting SAXS measurements during the elution of the target biomacromolecule. This technique has resulted in a paradigm shift in biomacromolecule solution scattering. Currently, the application of the SEC–SAXS system to small-angle neutron scattering (SANS) is being advanced. However, since the target biomacro­mol­ecules in the sample solution are not only purified but also diluted by SEC and pass through the sample cell in a short time, this method is being implemented in SANS instruments at high neutron flux. Here, we developed a new type of SEC–SANS system that can operate effectively with a SANS instrument at medium neutron flux. Its key innovation is the design and optimization of a dedicated flow path that allows for the storage of only the target biomolecules eluted from SEC in the sample cell. This innovation enables long-duration measurements, termed the ‘stopping mode’, for SEC samples. Consequently, this method allows for acquiring high-precision solution scattering data for target biomacromol­ecules, enabling SEC–SANS measurements even with SANS instruments at medium neutron flux.

## Abstract 2

Incorporating a size-exclusion chromatography–small-angle neutron scattering (SANS) system for a SANS instrument with medium neutron flux has resulted in the successful observation of the scattering profiles of target components in complexes.

## 1. Introduction

Biomacromolecules develop their biological functions in solution; hence, determining their solution structures offers clues for understanding their mechanisms. Recent instrument and sample environment improvements in small-angle scattering (SAS) methods have enabled the observation of biomacromolecule solution structures utilizing high signal-to-noise ratio scattering data (Bernadó *et al.*, 2018 ▸; Mahieu & Gabel, 2018 ▸; Trewhella, 2022 ▸). Furthermore, computational techniques such as molecular dynamics simulations have enabled the analysis of biomacromolecule solution structures with fluctuations in the scattering profile (Gräwert & Svergun, 2020 ▸; Matsumoto *et al.*, 2020 ▸; Shimizu *et al.*, 2022 ▸). As a result, SAS methods have recently played significant roles in the field of structural biology.

Since SAS observes the volume-fraction-weighted sum of all components in a solution, not only target proteins but also aggregates and/or degradates, in biomacromolecular solutions, obtaining the scattering profile of the target component is a prerequisite for accurately resolving the solution structure. Accordingly, small-angle X-ray scattering coupled with size-exclusion chromatography (SEC–SAXS) has enabled the selective observation of the scattering profiles of target components or complexes without aggregates or degradates in biomacromolecular solutions (Mathew *et al.*, 2004 ▸; David & Pérez, 2009 ▸; Bucciarelli *et al.*, 2018 ▸; Ryan *et al.*, 2018 ▸; Inoue *et al.*, 2019 ▸; Shih *et al.*, 2022 ▸). At present, SEC–SAXS is a conventional means of measurement, especially for bio­macro­molecules.

Another SAS method, small-angle neutron scattering (SANS), is also utilized for determining biomacromolecule solution structures. At the energies typically used for SAXS or SANS measurements, X-rays primarily interact with and are scattered by electrons, whereas neutrons predominantly interact with and are scattered by the nucleus. SANS offers different structural information from that of SAXS through the usage of contrast variation techniques, which exploit the difference in neutron scattering length between hydrogen and deuterium. Therefore, the complementary use of SAXS and SANS has allowed meaningful research analyzing biomacromolecular complexes (Lapinaite *et al.*, 2013 ▸; Matsumoto *et al.*, 2020 ▸; Yunoki *et al.*, 2022 ▸). Note that obtaining the SANS profile of target components without aggregates or degradates is essential for detailed structural analysis. Recently, SANS combined with SEC (SEC–SANS) has become a novel option for implementation in SANS instruments with high neutron flux (Johansen *et al.*, 2018 ▸; Martel *et al.*, 2023 ▸; Thomas *et al.*, 2024 ▸). As a next step, the SEC–SANS option is expected to be applied to SANS instruments with a medium neutron flux. However, since SEC–SANS measurements require a high-flux neutron beam due to the necessity of eluted sample solution flow, similar to SEC–SAXS, implementing the SEC–SANS option in SANS instruments with medium neutron flux is difficult because flowing sample solution significantly limits the measurement time of the target component in solution. Recently, a SEC–SAXS system optimized for laboratory-based SAXS instruments has been developed, wherein the photon flux is several orders lower than that at synchrotron facilities (Inoue *et al.*, 2019 ▸). The key technology adopted in the laboratory-based SEC–SAXS system is a ‘stopping mode’, which enables the continuous measurement of the most concentrated target component instead of the flowing sample solution. Using this mode, we selectively observed the scattering profile of the target component in a protein solution with a volume and concentration similar to those applied for the synchrotron-based SEC–SAXS system. Therefore, a SEC–SANS system with a ‘stopping mode’ is expected to achieve SEC–SANS measurements even with a SANS instrument with medium neutron flux.

In this work, we aimed to develop a SEC–SANS system equipped with a ‘stopping mode’ to fine-tune SANS instruments with medium neutron flux. The SEC–SANS system developed was assessed using several representative protein samples, and the feasibility of advanced measurements was demonstrated by coupling SEC–SANS with the inverse contrast matching (iCM) method (Sato *et al.*, 2021 ▸; Yunoki *et al.*, 2022 ▸) (SEC–iCM–SANS) for the partial structural analysis of a protein complex.

## 2. Materials and methods

### 2.1. Samples

Ovalbumin (OVA, A7641), bovine serum albumin (BSA, A3641) and apoferritin (AF, A4612) solutions purchased from Sigma–Aldrich (St Louis, MO, USA) were prepared as representatives of low-, medium- and high-molecular-mass proteins, respectively. These samples were used without further purification. Clock proteins KaiB and KaiC were also prepared to study protein complexes using SEC–iCM–SANS. KaiB and KaiC originating from *Synechococcus elongatus* PCC 7942 were expressed and purified as previously described (Oyama *et al.*, 2016 ▸; Sugiyama *et al.*, 2016 ▸; Murakami *et al.*, 2019 ▸). To prepare partially deuterated KaiB, the bacterial cells were grown in M9 minimal H_2_O/D_2_O mixture media containing hydrogenated and deuterated glucose (1,2,3,4,5,6,6-*d*7 and 98%; Cambridge Isotope Laboratories Inc., Tewksbury, MA, USA) as previously described (Okuda *et al.*, 2021 ▸). The degree of deuteration of KaiB was estimated to be 71% using MALDI-TOF (matrix-assisted laser desorption/ionization time of flight) mass spectrometry. The phospho­ryl­ation mimicking of KaiC, in which one phospho­rylation site (S431) was substituted with an aspartate residue (S431D), was employed. The purified KaiB and KaiC proteins were mixed at a molar ratio of 12:6 and incubated at 303 K for 48 h to promote the formation of the KaiB–KaiC (BC) complex (Simon *et al.*, 2022 ▸).

The eluents for OVA, BSA and AF were 99% D_2_O buffer containing 100 m*M* Tris–HCl (pH 7.5) and 100 m*M* NaCl. The eluents for clock protein solutions were 98% D_2_O buffer containing 50 m*M* sodium phosphate (pH 7.8), 150 m*M* sodium chloride, 5 m*M* magnesium chloride, 0.5 m*M* EDTA, 1 m*M* di­thio­threitol, 3 m*M* ATP, 50 m*M* glutamic acid and 50 m*M* arginine. The D_2_O/H_2_O ratio in the eluents was measured using Fourier transform infrared spectroscopy (FT-IR-4600; JASCO, Tokyo, Japan) as previously described (Okuda *et al.*, 2021 ▸). The injection concentrations for SEC–SANS measurements were 7.7 mg mL^−1^ for OVA, 7.9 mg mL^−1^ for BSA, 12.0 mg mL^−1^ for AF, 1.2 mg mL^−1^ for 71% deuterated KaiB (*71d*B), 5.6 mg mL^−1^ for hydrogenated KaiC (*h*C), 6.8 mg mL^−1^ for the mixture of *71d*B and *h*C, and 6.8 mg mL^−1^ for the mixture of hydrogenated KaiB (*h*B) and *h*C. The injection volume was set to 500 µL for all samples.

### 2.2. HPLC system and SEC columns

Prominence (SHIMADZU, Kyoto, Japan) comprising a controller (CBM-20A), solvent-delivery pump (LC-20Ai) and a UV detector (SPD20A) was utilized as the high-performance liquid chromatography (HPLC) system. A Superdex 200 Increase 10/300 GL column (Cytiva, Marlborough, MA, USA) was equipped upstream of the HPLC UV detector as a SEC column for the present measurements. The flow rate was set at 0.5 mL min^−1^ for SEC–SANS measurements.

### 2.3. SANS instrument

SANS measurements were performed using the SANS-U instrument (Okabe *et al.*, 2005 ▸; Mayumi *et al.*, 2024 ▸) at JRR-3 (Japan Atomic Energy Agency, JAEA, Ibaraki, Japan). The samples were irradiated with a neutron beam with a wavelength (λ) of 6.0 Å and wavelength distribution (Δλ/λ) of 10%. To maximize the neutron intensity at the sample position, the diameter of the beam was set to 15 mm for the present work. Scattered neutrons were recorded using a two-dimensional detector (Ordela, Oak Ridge, TN, USA). The sample-to-detector distances were set to 4000 and 1030 mm, covering the *q* range 0.01–0.3 Å^−1^. The source-to-sample distance (boron-coated collimator length) was set to 4000 mm. The neutron flux at the sample position was ≈ 6 × 10^5^ n cm^−2^ s^−1^, which was one to two orders of magnitude lower than that of the Bio-SANS instrument at Oak Ridge National Laboratory (Oak Ridge, TN, USA) (≈ 2 × 10^6^ n cm^−2^ s^−1^ at λ = 6.0 Å, Δλ/λ = 15% and collimator length ≈ 6000 mm) and the D22 instrument at the Institut Laue–Langevin (ILL, Grenoble, France) (≈ 3 × 10^7^ n cm^−2^ s^−1^ at λ = 6.0 Å, Δλ/λ = 10% and collimator length ≈ 5600 mm). Two-dimensional scattering patterns were converted to one-dimensional scattering profiles using the *Red2D* software (https://github.com/hurxl/Red2D). After correction by the transmittance and subtraction of buffer scattering, the scattering intensity was converted to an absolute scale using the standard scattering intensity of H_2_O (0.89 cm^−1^; Shibayama *et al.*, 2005 ▸). All measurements were performed at 298 K.

### 2.4. SANS cell

The flat inner cell was made of MACOR with quartz windows, each with a thickness of 1 mm. The inner cell has a disc-shaped sample space with a diameter of 18 mm and an optical path of 1 mm [volume: 254 µL; Fig. S1(*a*) of the supporting information]. For temperature control, the inner cell was embedded in a cell jacket equipped with flow channels to an external circulator. To avoid scattering from the SANS cell itself as much as possible, the SANS cell was sandwiched between cadmium plates with a 22 mm-diameter hole at the center [Fig. S1(*b*)].

To avoid a situation where the bubble remains inside the SANS cell, the top part of the cell was processed into a teardrop shape [Fig. S1(*a*)]. After the SEC–SANS experiment, the cell sample solution was collected using a needle and syringe. Subsequently, the concentration of the sample in the SANS cell was measured using a NanoDrop One UV Spectrometer (Thermo Fisher Scientific, Waltham, MA, USA).

### 2.5. UV–Vis–NIR unit

**Figure 1:**
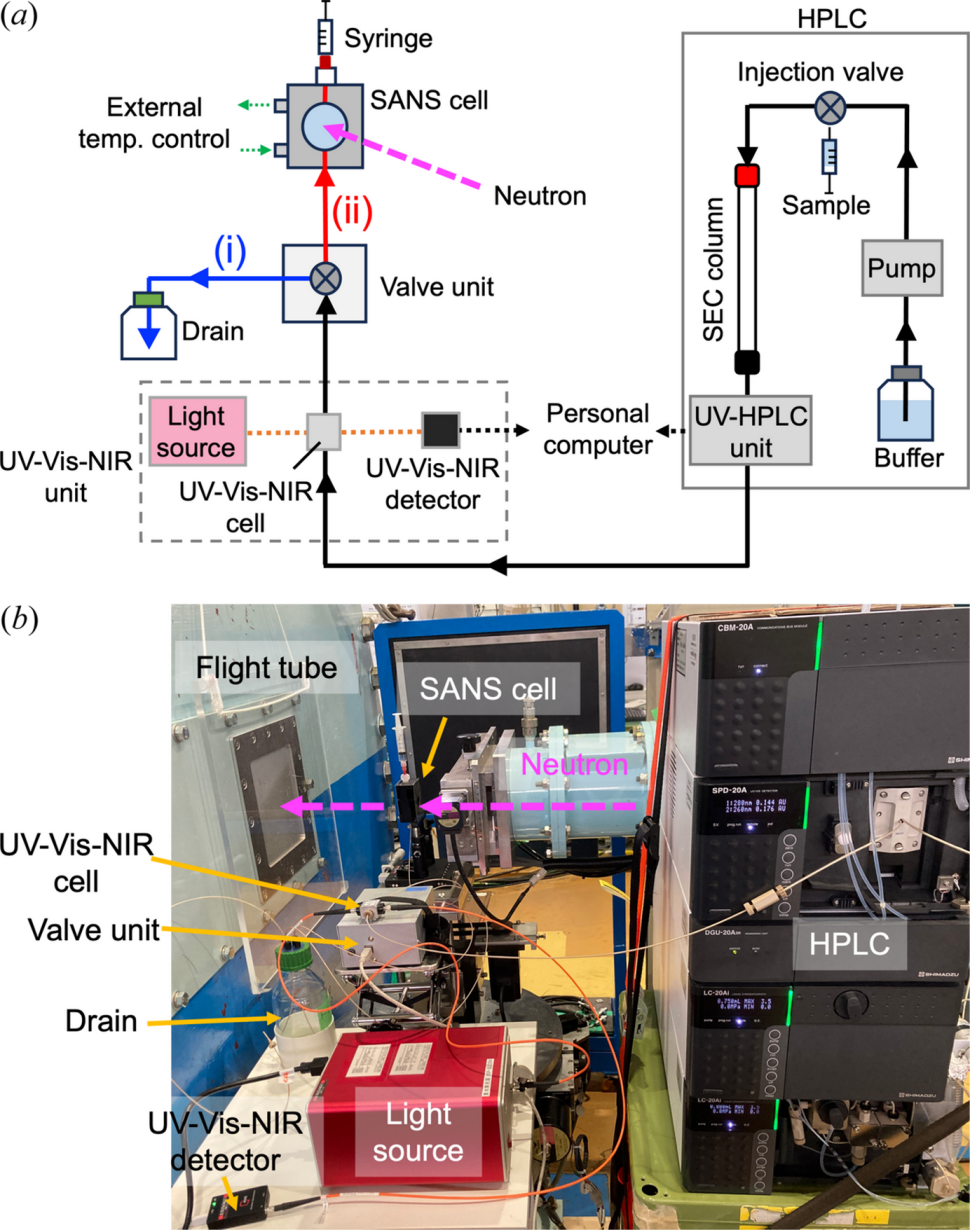
Overview of the SEC–SANS system. (*a*) Schematic of the flow route of the SEC–SANS system. Solid black arrows represent the flow route from the HPLC to the valve unit. Solid blue and solid red arrows represent the flow routes to (i) the drain and (ii) the SANS cell, respectively. The broken yellow line represents the optical fiber in the UV–Vis–NIR unit. Broken green arrows indicate the circular flow of the external temperature control. (*b*) SEC–SANS system installed in SANS-U.

To monitor sample elution near the SANS cell, a UV–Vis–NIR unit was installed upstream of the SANS cell [Fig. 1 ▸(*a*)]. A stabilized deuterium light source (THOLAB Inc., Newton, NJ, USA) that emits output in the 200–700 nm range, monitored using a Qmini2 UV compact detector (Broadcom Inc., Palo Alto, CA, USA), was used to evaluate integrated UV–Vis–NIR ranging from 190 to 1100 nm at a spectral resolution of 0.3 nm. The absorbance was measured using our UV–Vis–NIR cell (UNISOKU Co. Ltd, Osaka, Japan), which was connected to the deuterium light source and compact detector by optical fiber cables [Fig. S1(*c*)]. The optical path of the UV–Vis–NIR cell was set to 2 mm. The time course of absorbance covering the 200–350 nm wavelength range was monitored by the UV–Vis–NIR detector.

### 2.6. Flow-route control by a valve unit

Considering neutron flux at the sample position, SANS-U is categorized as a SANS instrument with medium neutron flux. Due to beam flux limitations, the SEC–SANS measurement of the flowing eluted sample solution using SANS-U is difficult. Therefore, a SEC–SANS system that enables the selective flow and measurement of the target component in the solution was developed. In addition to the UV–Vis–NIR unit near the SANS cell, an appropriate flow-route control system to the SANS cell should be installed in the SEC–SANS system. Considering these design concepts, the SEC–SANS system was constructed. Fig. 1 ▸(*b*) shows an overview of the SEC–SANS system installed in the SANS-U instrument. The SANS cell was positioned 60 mm from the sample aperture on the detector side. The valve unit (UNISOKU Co. Ltd) [Fig. S1(*d*)], located between the SANS cell and UV–Vis–NIR unit [Fig. 1 ▸(*a*)], plays a significant role in the ‘stopping mode’ of our SEC–SANS system. Specifically, SEC–SANS measurements were performed by switching the flow route using the valve unit as follows:

(1) When a sample solution was injected into the HPLC system, the flow route was set to a drain bottle [flow route (i) in Fig. 1 ▸(*a*)].

(2) The absorbance of the eluted sample from the SEC column was monitored using the UV–Vis–NIR unit. Before eluting the target component in the sample solution, the flow route was maintained as route (i) (to the drain bottle). Specifically, the eluted sample solution did not flow into the SANS cell.

(3) When the target component absorbance approximated the maximum, the eluted sample solution was loaded into the SANS cell by setting the flow route to (ii) in Fig. 1 ▸(*a*).

(4) After confirming that the interior of the SANS cell was filled with the target component in the sample solution, the flow route was switched from (ii) to (i). This is referred to as the ‘stopping mode’.

### 2.7. SEC–SANS measurement

The SANS measurement of a buffer for subtraction from a sample solution was performed as follows. After the equilibration of the SEC column with a three-column volume of running buffer, we changed the flow route from the drain bottle [flow route (i) in Fig. 1 ▸(*a*)] to the SANS cell [flow route to (ii) in Fig. 1 ▸(*a*)]. After confirming that the SANS cell was filled with the buffer, we then changed the flow path to the SANS cell [flow route (ii)] to the drain bottle [flow route (i)]. Prior to the scattering measurement, we measured transmission of the buffer to confirm the absence of contamination with hydrogen atoms, especially H_2_O. We then performed a scattering measurement of the buffer.

After the buffer measurement, the SANS measurement for the sample solutions commenced following procedures (1)–(4) in the preceding section. In the present work, all the sample solutions have already been exchanged with D_2_O buffer prior to the injection into the SEC column. Hence, buffer subtraction worked appropriately despite the different SEC runs between buffer and sample measurements. The duration of each measurement was fixed at 10 min, and the measurements were accumulated until sufficient counting statistics were obtained. When the errors of *R*_g_ or *I*(0) values are less than 3%, we judged that sufficient counting statistics were obtained. The time progression of SANS profiles was monitored to determine re-aggregation and degradation during measurement. When a SANS profile was unchanged during the entire measurement time, we simply averaged all observed SANS profiles. In cases where changes in the SANS profiles were observed, the profiles were averaged, excluding those with detected changes.

### 2.8. Analytical ultracentrifugation measurement

Sedimentation velocity-analytical ultracentrifugation (AUC) measurements were conducted using ProteomeLab XL-I (Beckman Coulter, Brea, CA, USA). The samples were loaded into cells equipped with 12 mm-path-length aluminium centerpieces. All measurements were performed using Rayleigh interference optics at 298 K. The rotor speed was set at 45000 rev min^−1^. The time evolution of the sedimentation data was analyzed using the multi-component Lamm equation (Lebowitz *et al.*, 2002 ▸). The weight-concentration distribution *c*(*s*_20,w_) as a function of the sedimentation coefficient and frictional ratio *f*/*f*_0_ was computed using the *SEDFIT* software (version 15.01c; Schuck, 2000 ▸). The sedimentation coefficient *s*_20,w_ was normalized with respect to the value at 293 K in pure water.

### 2.9. AUC–SANS treatment

As a reference, an aggregation-free scattering profile was obtained by the AUC–SANS treatment of a normal SANS measurement scattering profile. The details of the AUC–SANS treatment have been reported in our previous study (Morishima *et al.*, 2020 ▸, 2023 ▸). AUC and SANS measurements were performed for OVA, BSA and AF at 3.1, 3.1 and 3.8 mg mL^−1^, respectively.

## 3. Results and discussion

### 3.1. Separation resolution of the UV–Vis–NIR unit

**Figure 2:**
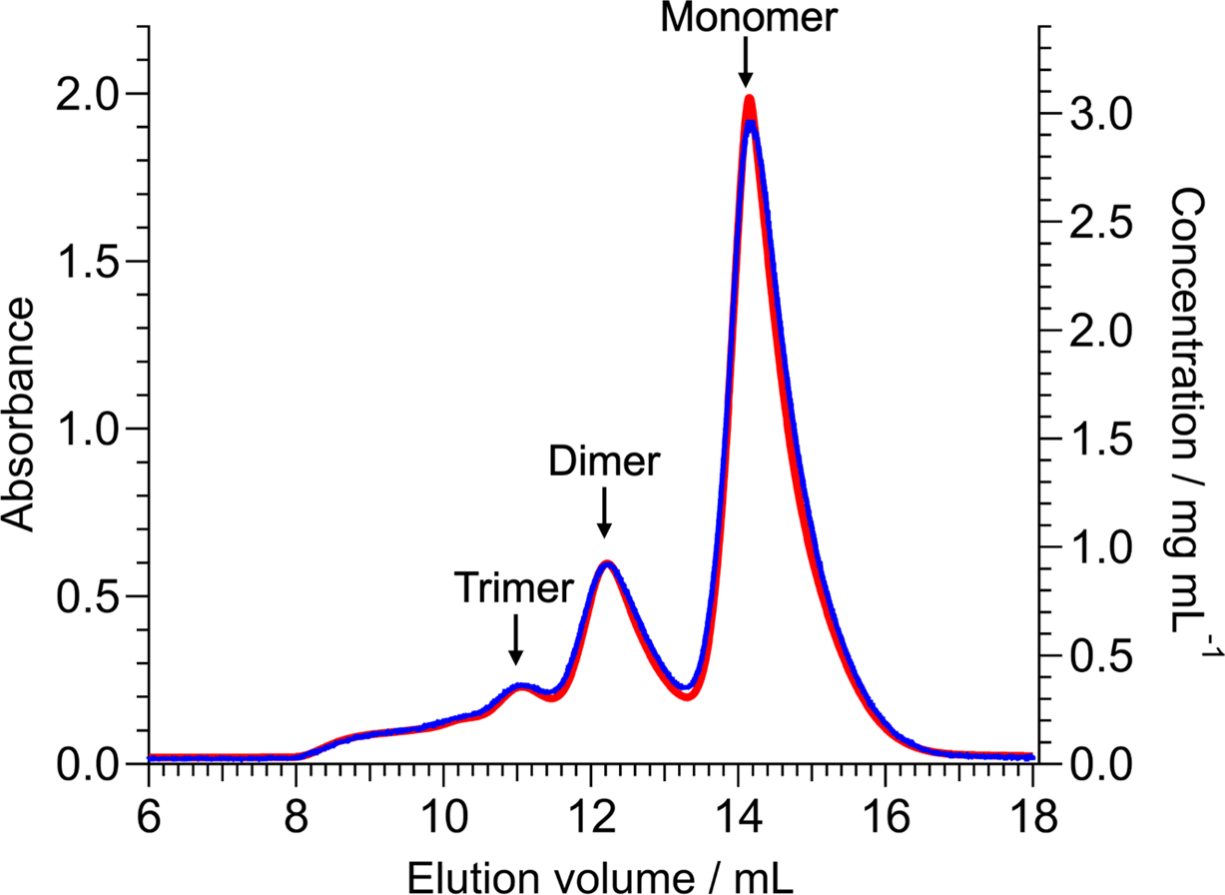
Separation resolution of UV–HPLC and UV–Vis–NIR units. Red and blue lines represent the SEC charts (wavelength = 280 nm) for the unpurified BSA solution for the UV–HPLC and UV–Vis–NIR units, respectively. The elution volume of the UV–Vis–NIR unit is horizontally shifted, considering the dead volume between the UV–HPLC and UV–Vis–NIR units.

The SEC chart of the UV–Vis–NIR unit was used to determine the timing for target sample flow into the SEC–SANS cell (refer to Fig. 1 ▸). Note that large volumes due to the length and/or thickness of the tube from the HPLC outlet to the UV–Vis–NIR unit could deteriorate the separation resolution of the eluted sample at the cell position. To avoid this problem, the tubing length from the HPLC outlet to the UV–Vis–NIR unit (750 mm) and the inner diameter of the PEEK tube (0.13 mm) were minimized. Fig. 2 ▸ shows the SEC charts of the unpurified BSA solution from the UV–Vis–NIR and UV–HPLC units. The peak positions of the BSA monomers and oligomers exhibited good agreement between the two SEC charts. To evaluate monomer, dimer and trimer peak resolutions, we fitted the elution volume dependence of UV–HPLC and UV–Vis–NIR unit absorbances with the sum of three Gaussians. The full width at half-maximum (FWHM) values of each peak from the UV–HPLC and UV–Vis–NIR units are summarized in Table 1 ▸. Compared with the FWHM values calculated from UV–HPLC, those from the UV–Vis–NIR unit were slightly increased. To assess whether or not this deterioration of the separation resolution of the eluted sample non-negligibly affects the practical use of SEC–SANS, we also evaluated the separation resolution of the SANS cell in the next section.

### 3.2. Separation resolution at SANS cell

**Figure 3:**
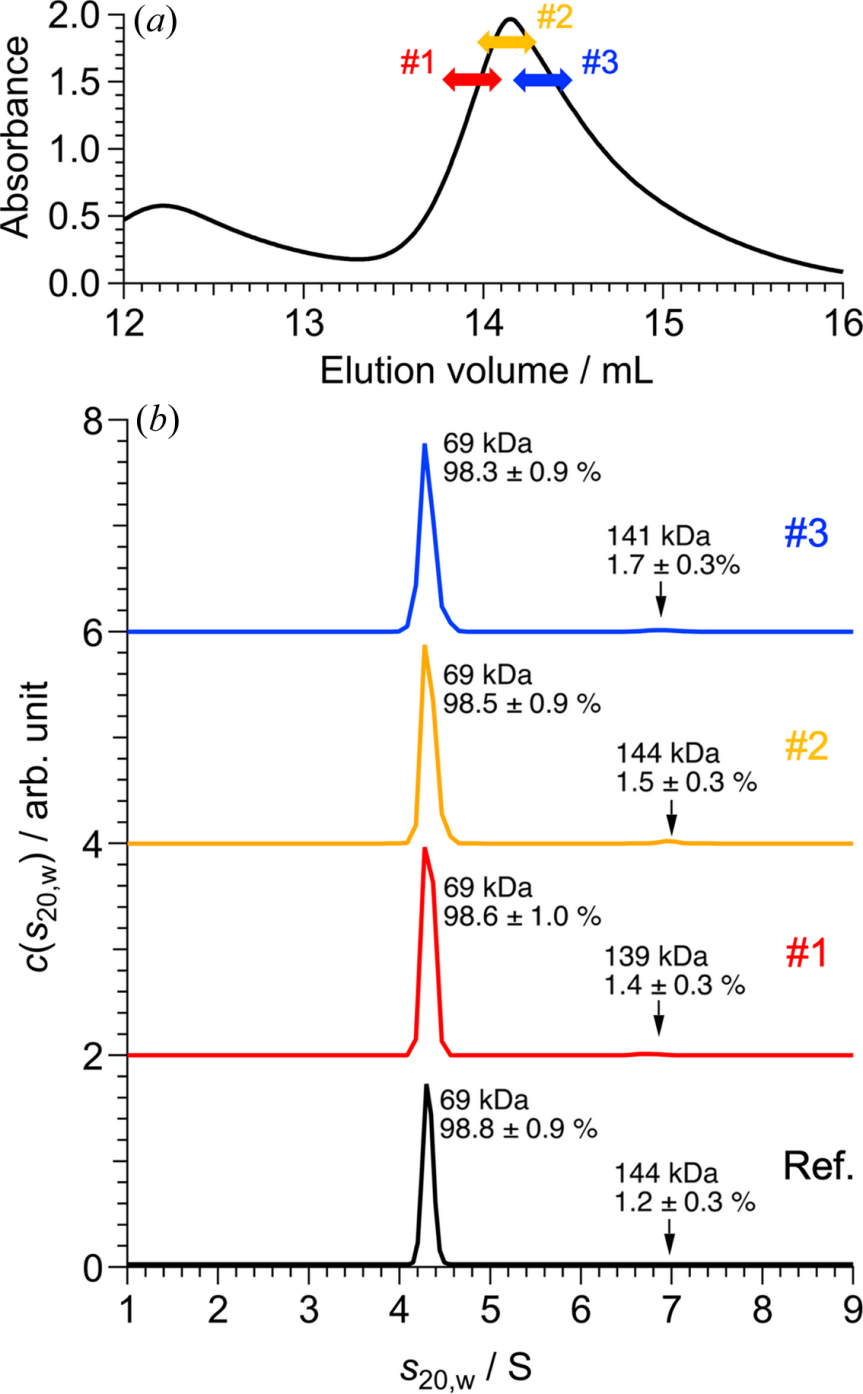
Separation resolution at the SANS cell. (*a*) SEC chart for the unpurified BSA solution measured using a UV–Vis–NIR unit at a wavelength of 280 nm. Red, yellow and blue arrows represent the elution volume ranges at which the eluted samples were loaded into the SANS cell. The elution volumes of each fraction were (1) 13.8–14.1 mL, (2) 14.0–14.3 mL and (3) 14.2–14.5 mL. (*b*) Red, yellow and blue lines indicate the AUC results for fractions #1, #2 and #3, respectively. The black line shows the AUC result for monomeric BSA immediately after standard SEC purification with the same column. The molecular mass values calculated from the sedimentation coefficient and peak area ratio for each peak are included in (*b*).

Compared with the cell volume (140 µL) of the SEC-SANS system installed at D22 in ILL, the cell volume in our system (254 µL) is relatively large. In addition to the dead volume between the HPLC and UV–Vis–NIR units, such a large cell volume could influence the separation resolution of the eluted sample in the cell. Next, a loading test on the SANS cell was performed utilizing unpurified BSA as one of the representative polydispersity samples. A monomeric BSA fraction was loaded into the SANS cell at the three different elution volume ranges shown by the colored arrows in Fig. 3 ▸(*a*). Subsequently, the monomeric BSA fraction was collected from the SANS cell, and AUC measurements were conducted for these solutions. The distributions [*c*(*s*_20,w_)] of the normalized sedimentation coefficients (*s*_20,w_) of the three solutions are summarized in Fig. 3 ▸(*b*) and Table S1 of the supporting information. The weight fraction of dimeric BSA was less than 2% for all three solutions, indicating that the differences between the three sample solutions were within the experimental error range. The dimeric BSA contamination levels were almost the same as those for monomeric BSA, even immediately after standard SEC purification using the same SEC column [black line in Fig. 3 ▸(*b*)]. The SEC separation resolution was confirmed to be preserved at the cell position.

### 3.3. SEC–SANS measurement of proteins with various molecular mass

**Figure 4:**
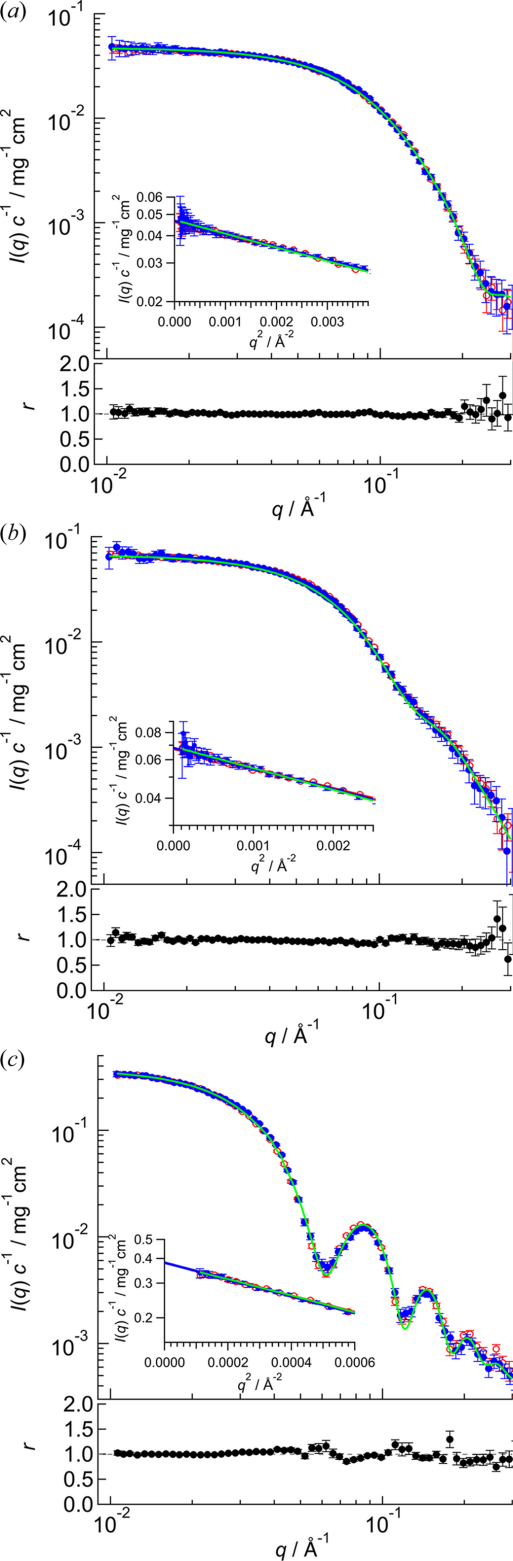
SANS profiles of (*a*) OVA, (*b*) BSA and (*c*) AF. Blue and red circles in the upper panels show the concentration-normalized SANS profiles obtained using SEC–SANS and AUC–SANS, respectively. Solid green lines indicate the calculated scattering profile from the crystal structures [PDB entries 1ova (Stein *et al.*, 1991 ▸), 4f5s (Bujacz, 2012 ▸) and 4v1w (Russo & Passmore (2014 ▸) for OVA, BSA and AF, respectively]. Insets represent their Guinier plots. Solid blue and solid red lines in the Guinier plots indicate least-squares fitting using the Guinier formula for SEC–SANS and AUC–SANS results, respectively. Lower panels represent the ratio (*r*) of SEC–SANS to AUC–SANS scattering profiles.

SEC–SANS measurements were performed for OVA, BSA and AF as representatives of low-, medium- and high mol­ecular mass proteins, respectively. The SEC charts for OVA, BSA and AF are shown in Figs. S2(*a*), S2(*b*) and S2(*c*), respectively. The sample flowed into the SANS cell at the elution volume highlighted in the SEC charts of Fig. S2. Total measurement times are summarized in Table S2. During the SANS measurement, we confirmed the absence of re-aggregation and degradation through the time dependences of the gyration radius (*R*_g_) and forward scattering intensity [*I*(0)] (refer to Fig. S3). The scattering profiles obtained from the SEC–SANS system for OVA, BSA and AF are shown in Figs. 4 ▸(*a*), 4(*b*) and 4(*c*), respectively. *R*_g_ and concentration-normalized forward scattering intensity [*I*(0)*c*^−1^] values are summarized in Table 2 ▸. As indicated in Section 2.4, the weight concentration (*c*) of each sample was measured with the solution collected from the SANS cell after the measurement (Table S2). To check for contamination such as aggregates and degradates in the scattering profile, the SEC–SANS scattering profiles were compared with those of the AUC–SANS method [red circles in Figs. 4 ▸(*a*), 4 ▸(*b*) and 4 ▸(*c*)]. AUC–SANS derives the aggregation-free scattering profile using the SANS profile and AUC weight fraction distribution (Morishima *et al.*, 2020 ▸, 2023 ▸). *I*(*q*), *I*(0)*c*^−1^ and *R*_g_ exhibited good agreement between SEC–SANS and AUC–SANS (Fig. 4 ▸ and Table 2 ▸). The remaining BSA dimer with a small weight fraction (≈ 2%) was confirmed to negligibly affect the scattering profile of the BSA monomer (refer to Fig. S4, Table S3). Thus, the collective evidence proves that our SEC–SANS system derives reliable scattering profiles for proteins with various molecular masses from solutions containing aggregates and degradates (Fig. S2). From two different approaches, we succeeded in obtaining SANS profiles from the target component without inconsistency. To utilize AUC–SAS appropriately, the following two prerequisites should be fulfilled. Firstly, the weight fraction of a target component should be higher than 0.8. Secondly, huge aggregates (association number > 5) and degradates should not coexist in the solution. Hence, a sample that cannot satisfy the above two prerequisites could be a target for SEC–SANS. To be more specific, if the target molecule is a multi-component system, it is possible to selectively obtain the scattering profile of the target component through the adoption of a ‘stopping mode’.

### 3.4. SEC–iCM–SANS measurements for clock protein complex

**Figure 5:**
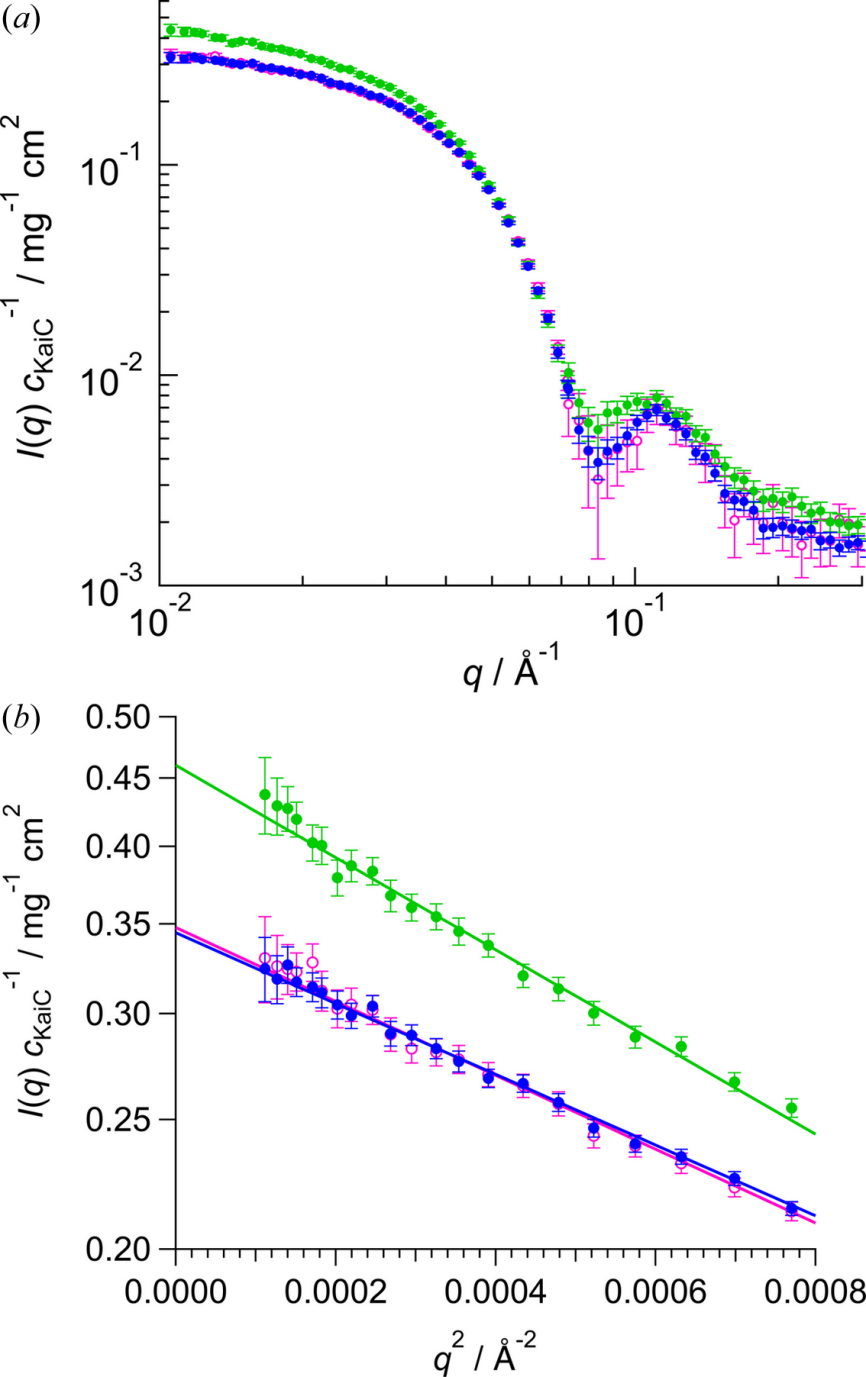
SEC–iCM–SANS measurement results for clock protein BC complex and KaiC. (*a*) Closed blue, closed green and open magenta circles show the SANS profiles for *71d*B*h*C, *h*B*h*C and *h*C, respectively. Scattering intensity was normalized by KaiC concentration (*c*_KaiC_). (*b*) Guinier plots of the scattering data as shown in (*a*). Solid lines correspond to the results of the least-squares fitting with the Guinier formula.

One of the noticeable characteristics associated with SANS is that biomacromolecules can become ‘scatteringly invisible’ by matching their scattering length density to that of the solvent, known as contrast matching (CM) and inverse contrast matching (iCM) (Sugiyama *et al.*, 2014 ▸). By exploiting this property, SANS makes it possible to selectively observe the partial structure of complexes or multi-component systems. Therefore, the SEC–SANS system developed was applied to the structural analysis of the partial structure of KaiC in the clock protein BC complex (Sugiyama *et al.*, 2016 ▸; Simon *et al.*, 2022 ▸). Specifically, we performed SEC–iCM–SANS measurements of the clock protein complex with stopping mode. For this purpose, a BC complex (*71d*B*h*C) comprising the scatteringly invisible component 71d-KaiB (*71d*B; refer to Fig. S5) and the visible component h-KaiC (*h*C) in 98% D_2_O buffer was prepared. In addition to the BC complex, aggregates and free KaiB coexisted in the mixed KaiB and KaiC solution [Figs. S6(*a*) and S6(*b*)]. SEC–iCM–SANS measurements were carried out for the BC complex fraction (highlighted region in Fig. S6). From the AUC measurement (Fig. S7), we confirmed that the BC complex (*71d*B*h*C) did not show dissociation and aggregation by the separation and dilution using SEC. The partial scattering profile of *h*C in *71d*B*h*C observed by SEC–iCM–SANS is shown by the blue circles in Fig. 5 ▸. As reference data, the SEC–SANS results for *h*C alone and the BC complex (*h*B*h*C) consisting of h-KaiB (*h*B) and *h*C are also shown as magenta and green circles, respectively. Table 3 ▸ presents *R*_g_ and 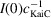, where 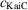 is the concentration of KaiC. The 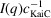, *R*_g_ and 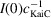 of *71d*B*h*C coincided with those of *h*C alone. Although a previous study suggested that the ring structure of KaiC is compressed to allow binding with KaiB (Swan *et al.*, 2022 ▸), no significant structural modulation of KaiC due to BC complex formation was observed in the present results. Our developed SEC–SANS system was experimentally confirmed to selectively determine the partial structure in a complex without aggregates and free components. Specifically, we demonstrated the feasibility of SEC–iCM–SANS measurements using a SANS instrument with medium neutron flux.

## 4. Conclusions

Using key ‘stopping mode’ technology, a SEC–SANS system was implemented in a SANS instrument with medium neutron flux, and we successfully observed the scattering profiles of target components in complexes in realistic measurement times. Since SEC–SANS is becoming a standard option for SANS instruments at neutron facilities around the world, our SEC–SANS system is expected to be adopted at other SANS instruments with medium neutron flux. The ‘stopping mode’ is also beneficial for measuring dilute or low-scattering contrast samples, even using SANS instruments with high neutron flux. Our system will contribute to the selective observation of target biomacromolecular complexes in multi-component and polydisperse solutions under physiological conditions, enabling further detailed analyses of biomacromolecular solution structure. Recently it was reported that the incorporation of the refractive index monitor into SEC–SAXS was advantageous for studying membrane proteins stabilized by detergents (Shih *et al.*, 2022 ▸). Hence, installation of other optional apparatus into our SEC–SANS system could enhance the applicability for further complex systems.

## Supplementary Material

Supporting figures and tables. DOI: 10.1107/S1600576725000779/tu5062sup1.pdf

## Figures and Tables

**Table 1:** FWHM values for the elution peaks corresponding to BSA monomers, dimers and trimers in SEC charts for the UV–Vis–NIR and UV–HPLC units The error corresponds to the standard deviation given by Gaussian curve fitting.

	FWHM (mL)	
	UV–Vis–NIR unit	UV–HPLC unit
Monomer	1.61 ± 0.01	1.48 ± 0.02
Dimer	1.81 ± 0.01	1.54 ± 0.01
Trimer	2.19 ± 0.01	1.71 ± 0.01

**Table 2:** Gyration radii (*R*_g_), concentration-normalized forward scattering intensities [*I*(0)*c*^−1^] and χ^2^ values between the experimental scattering profile and the calculated profile for the crystal structure of OVA, BSA and AF obtained using SEC–SANS and AUC–SANS

	SEC–SANS			AUC–SANS		
Sample	*R*_g_ (Å)	*I*(0)*c*^−1^ (mg^−1^ cm^2^)	χ^2^	*R*_g_ (Å)	*I*(0)*c*^−1^ (mg^−1^ cm^2^)	χ^2^
OVA	20.3 ± 0.3	0.0467 ± 0.0004	1.5	20.0 ± 0.2	0.0463 ± 0.0003	2.0
BSA	25.6 ± 0.6	0.068 ± 0.002	1.3	25.2 ± 0.2	0.067 ± 0.001	1.5
AF	54.4 ± 0.8	0.382 ± 0.005	2.0	53.8 ± 0.9	0.381 ± 0.004	2.3

**Table 3:** Gyration radii (*R*_g_), KaiC concentration normalized forward scattering intensities 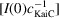 and molecular mass (*M*) calculated from *I*(0) for *h*C, *71d*B*h*C and *h*B*h*C, respectively

Sample	*R*_g_ (Å)	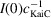 (mg^−1^ cm^2^)	*M* (kDa)
*h*C	43.7 ± 0.7	0.348 ± 0.004	334 ± 11
*71d*B*h*C	43.2 ± 0.6	0.346 ± 0.003	333 ± 10
*h*B*h*C	49.1 ± 0.9	0.460 ± 0.006	442 ± 14

## Data Availability

Details of samples, data collection and analysis in this study are summarized in Table S4. Any other data in this article are available from the authors upon reasonable request.
