# Supplementary material for: Size-exclusion chromatography–small-angle neutron scattering system optimized for an instrument with medium neutron flux
Source: J Appl Crystallogr. 2025 Feb 11;58(Pt 2):595–602. doi: 10.1107/S1600576725000779 (PMC11957415; doi:10.1107/S1600576725000779)
Supplement: Supplementary file 1 [file j-58-00595-sup1.pdf]

JOURNAL OF APPLIED CRYSTALLOGRAPHY

**Volume 58 (2025)**

**Supporting information for article:**

# Size exclusion chromatography–small-angle neutron scattering system optimized for an instrument with medium neutron flux

**Ken Morishima, Rintaro Inoue, Tatsuo Nakagawa, Masahiro Shimizu, Ritsuki Sakamoto, Tatsuro Oda, Koichi Mayumi and Masaaki Sugiyama**

# Supporting information

## Size exclusion chromatography-small-angle neutron scattering system optimized for an instrument with medium neutron flux

**Ken Morishima[a1], Rintaro Inoue[a1], Tatsuo Nakagawa[b], Masahiro Shimizu[a], Ritsuki Sakamoto[c], Tatsuro Oda[d], Koichi Mayumi[d] and Masaaki Sugiyama[a]***

[a]Institute for Integrated Radiation and Nuclear Science, Kyoto University, Kumatori, Sennan-gun, Osaka, 590-0494, Japan

[b] Unisoku Co. Ltd, 2-4-3 Kasugano, Hirakata, Osaka, 573-0131, Japan

[c]Graduate School of Science, Kyoto University, Kitashirakawa, Sakyo-ku, Kyoto, 606-8502, Japan

[d]The Institute for Solid State Physics, University of Tokyo, 5-1-5 Kashiwanoha, Kashiwa, Chiba, 277-8581, Japan

*Correspondence email: sugiyama.masaaki.5n@kyoto-u.ac.jp

[1]Ken Morishima and Rintaro Inoue contributed equally to this work.

## S1. Components of the SEC-SANS System

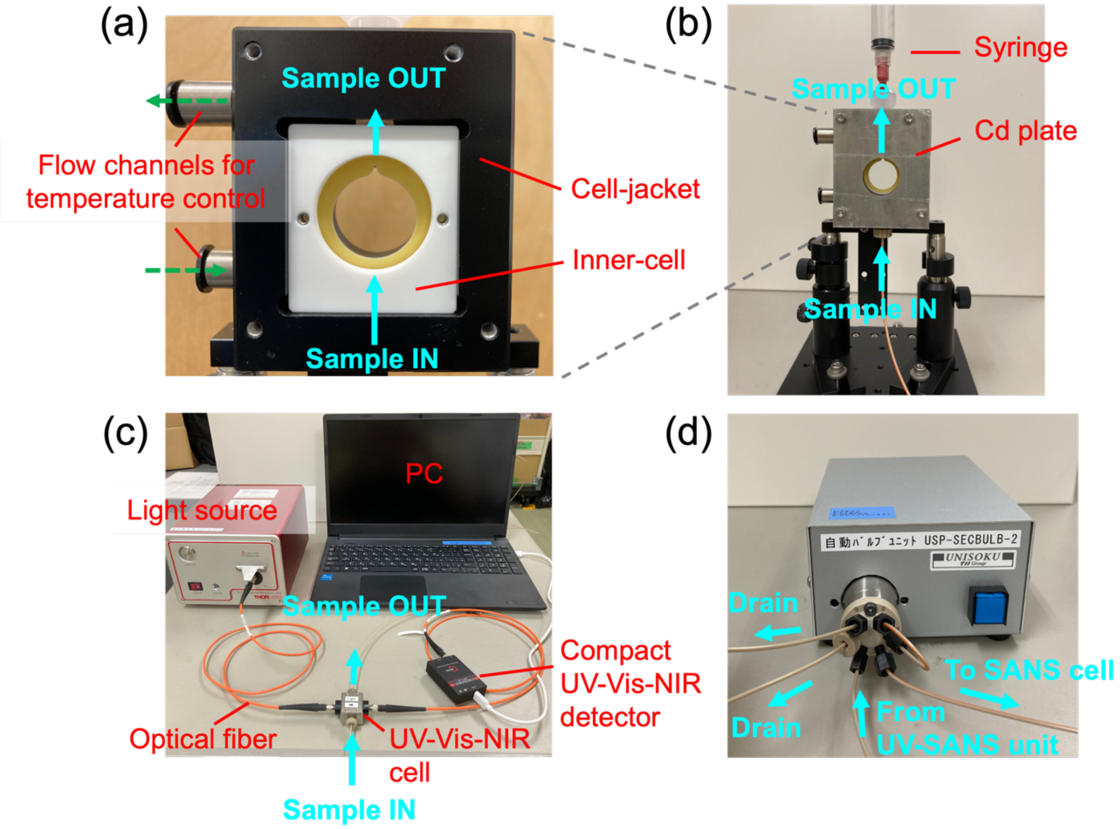


**Figure S1.** (a) SANS cell interior. The flat inner cell is embedded in a cell jacket. The sample is loaded into the cell from bottom to top. (b) Overview of SANS cell sandwiched by cadmium plates to avoid scattering from the cell jacket itself. The cell jacket has a tapered opening with a 22-mm outside diameter. (c) UV-Vis-NIR unit comprising a source light, UV-Vis-NIR cell, and compact UV-Vis-NIR detector. (d) Valve unit to switch the flow route (refer to Fig. 1 in main text).

## S2. Distribution of components in BSA solutions

**Table S1.** Sedimentation coefficient ($s_{20,w}$), molecular mass ($M$), and weight fraction ($w$) of monomers and dimers in the BSA solutions loaded into the SANS cell. Fractions are defined as those in Fig. 3.

Fraction #1

| $s_{20,w}$ / S | $M$ / kDa | $w$ / % |
|---|---|---|
| 4.3 | 69 | 98.3 ± 0.9 |
| 6.8 | 141 | 1.7 ± 0.3 |

Fraction #2

| $s_{20,w}$ / S | $M$ / kDa | $w$ / % |
|---|---|---|
| 4.3 | 69 | 98.5 ± 0.9 |
| 6.9 | 144 | 1.5 ± 0.3 |

Fraction #3

| $s_{20,w}$ / S | $M$ / kDa | $w$ / % |
|---|---|---|
| 4.3 | 69 | 98.6 ± 1.0 |
| 6.8 | 139 | 1.4 ± 0.3 |

Reference

| $s_{20,w}$ / S | $M$ / kDa | $w$ / % |
|---|---|---|
| 4.3 | 69 | 98.8 ± 0.9 |
| 6.9 | 144 | 1.2 ± 0.3 |

## S3. SEC Charts of OVA, BSA, and AF

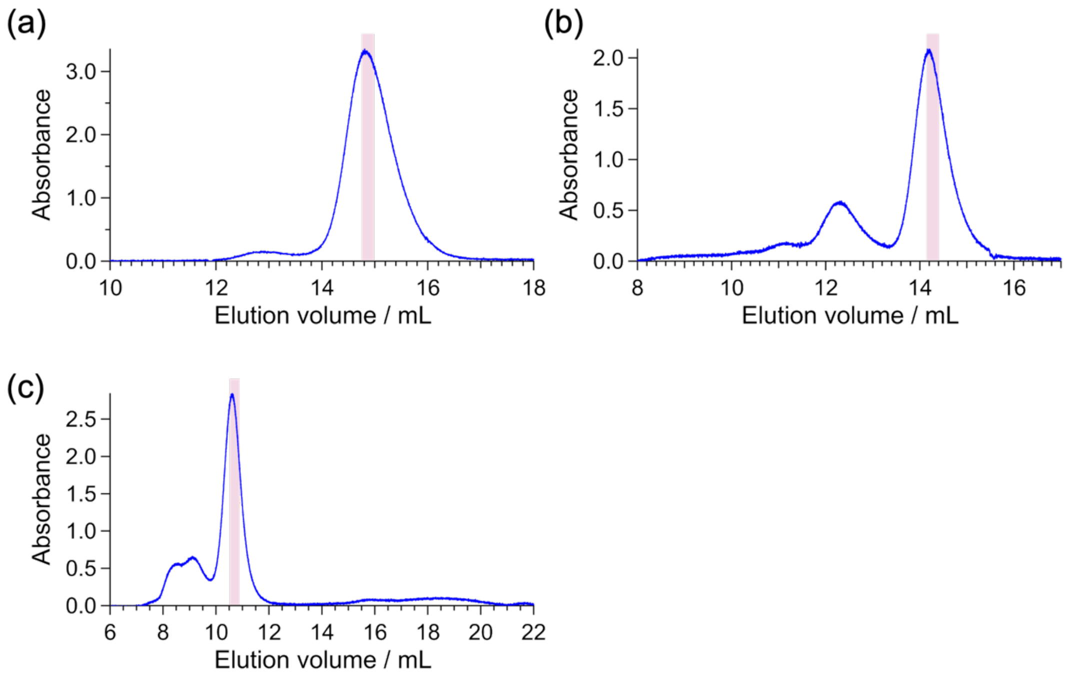


**Figure S2.** SEC charts of (a) OVA, (b) BSA, and (c) AF solutions for SEC-SANS measurements. The time evolution of absorbance at a wavelength of 280 nm was recorded with a UV-Vis-NIR unit. The pink highlighted area indicates the elution volume range at which the sample was loaded into the SANS cell. OVA, ovalbumin; BSA, bovine serum albumin; AF, apoferritin.

## S4. Measurement Time and Concentration in the SANS Cell

**Table S2.** Concentrations (*c*) loaded into the SANS cell and total measurement times ($t_m$) for OVA, BSA, AF, *71d*B, *h*C, *71d*B + *h*C, and *h*B + *h*C solutions.

| Sample | $t_m$ / min (SDD = 4m) | $t_m$ / min (SDD = 1m) | $c$ / mg mL$^{-1}$ |
|---|---|---|---|
| OVA | 240 | 240 | 2.1 |
| BSA | 360 | 240 | 2.0 |
| AF | 240 | 240 | 2.5 |
| *71d*B | 120 | 120 | 0.6 |
| *h*C | 240 | 240 | 1.1 |
| *71d*B + *h*C | 240 | 240 | 1.9 |
| *h*B + *h*C | 120 | 120 | 1.5 |

SDD, sample-to-detector distance; OVA, ovalbumin; BSA, bovine serum albumin; AF, apoferritin.

**S5. Time dependence of *I*(0) and $R_g$ during SANS measurement.**

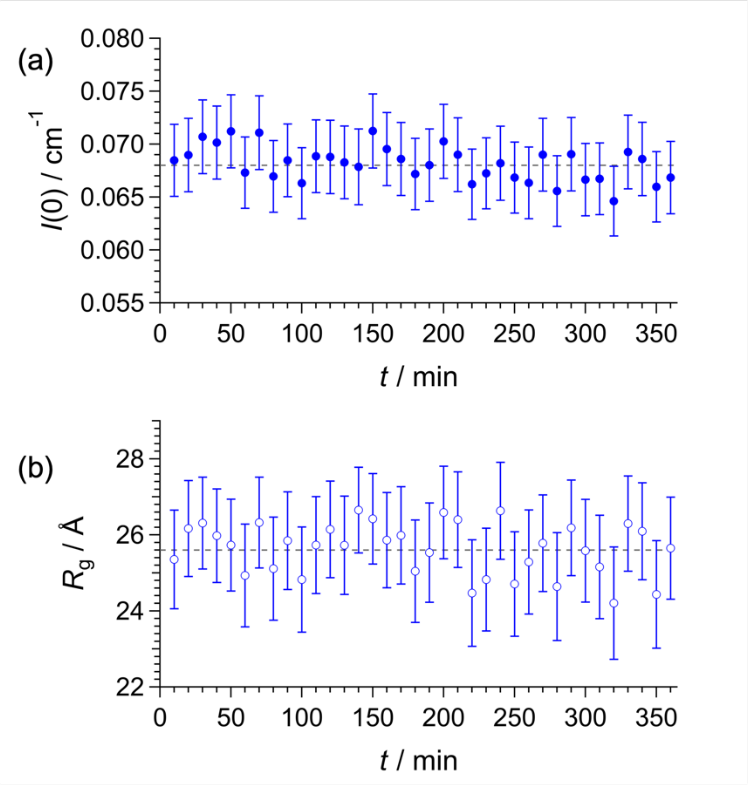


**Figure S3.** (a) Time dependences of $I(0)$ (a) and $R_g$ (b) for monomeric BSA solution.

## S6. Effect of the BSA Dimer with a Small Weight Fraction Remaining in the SANS Cell

To examine the effect of the remaining dimer on the SEC-SANS profile of BSA monomers, we performed AUC-SANS treatment of the scattering profile of BSA obtained by SEC-SANS [Fig. 4(b) in the main text]. Specifically, we additionally removed the contribution of the remaining BSA dimer (weight fraction = 1.7%) from the scattering profile obtained by SEC-SANS using AUC-SANS treatment. The blue and red circles in Fig. S3 show the scattering profiles obtained by SEC-SANS and following AUC-SANS treatment, respectively. Gyration radii ($R_g$) and concentration-normalized forward scattering intensities ($I(0)c^{-1}$) obtained by Guinier analysis are summarized in Table S3. The differences in the scattering profiles, $R_g$, and $I(0)c^{-1}$ before and after additional AUC-SANS treatment were within the experimental error range, showing the negligible effect of the remaining BSA dimers on the scattering profile of BSA monomers.

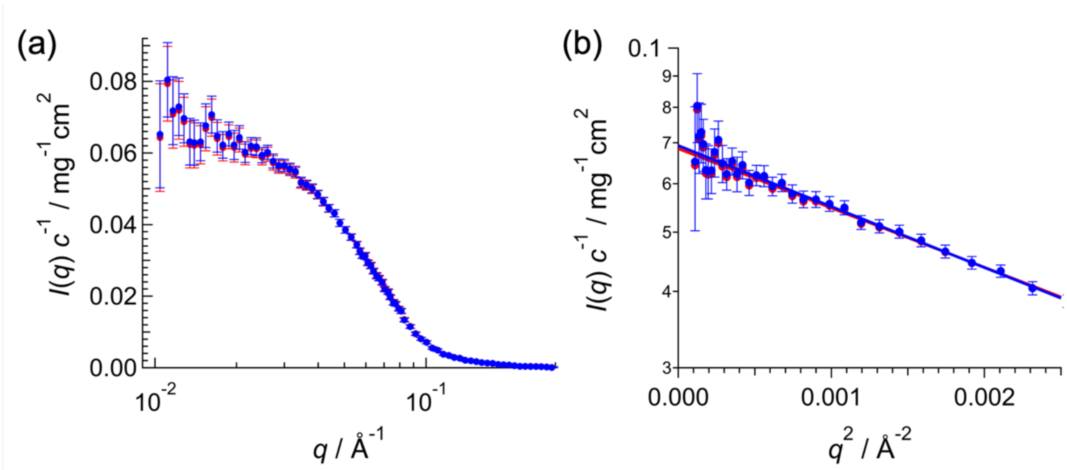


**Figure S4.** (a) Blue circles show the concentration-normalized SANS profile obtained by SEC-SANS [identical to Fig. 4(b) in the main text]. Red circles show the profile following additional AUC-SANS treatment of the profile obtained by SEC-SANS assuming BSA dimers with a 1.7% weight fraction. (b) Guinier plots and the least-squares fitting line with the Guinier formula.

**Table S3.** Gyration radii ($R_g$) and concentration-normalized forward scattering intensities ($I(0)c^{-1}$) of BSA for the scattering profiles obtained by SEC-SANS and additional AUC-SANS treatment.

| SEC-SANS | | Additional AUC-SANS treatment | |
|---|---|---|---|
| $R_g$ / Å | $I(0)c^{-1}$ / mg$^{-1}$ cm$^2$ | $R_g$ / Å | $I(0)c^{-1}$ / mg$^{-1}$ cm$^2$ |
| 25.6 ± 0.6 | 0.068 ± 0.002 | 25.4 ± 0.6 | 0.069 ± 0.002 |

## S7. SEC-iCM-SANS Measurements for Clock Proteins

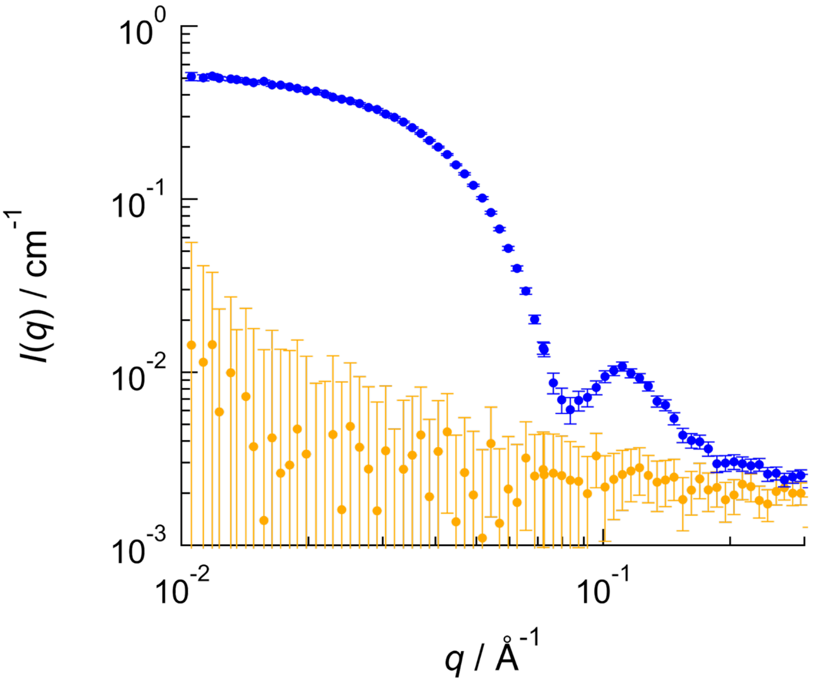


**Figure S5.** Closed blue and yellow circles show the SANS profiles obtained by the SEC-iCM-SANS of the *71d*B*h*C complex and *71d*B, respectively.

## S8. SEC Charts of Clock Protein Solutions

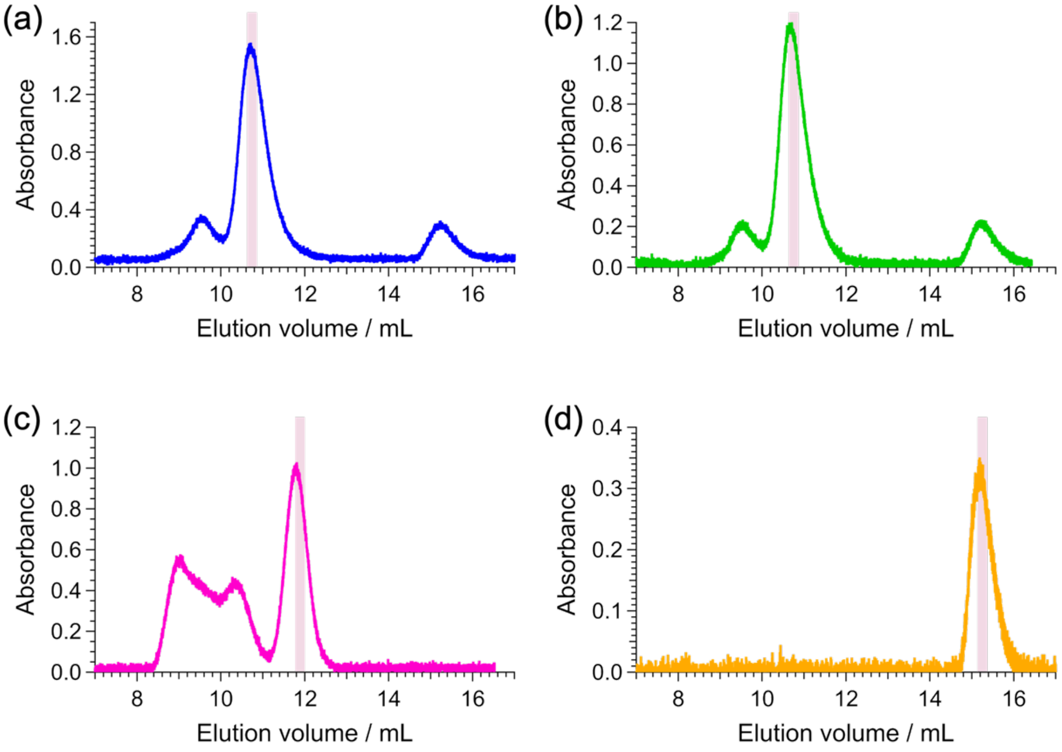


**Figure S6.** SEC charts of (a) a mixed *71d*B and *h*C solution, (b) mixed *h*B and *h*C solution, (c) *h*C solution, and (d) *71d*B solution. The time evolution of absorbance at a wavelength of 285 nm was recorded using a UV-Vis-NIR unit. The pink highlighted area indicates the elution volume at which the sample was loaded into the SANS cell.

**S9. AUC measurement for BC complex after SEC-SANS.**

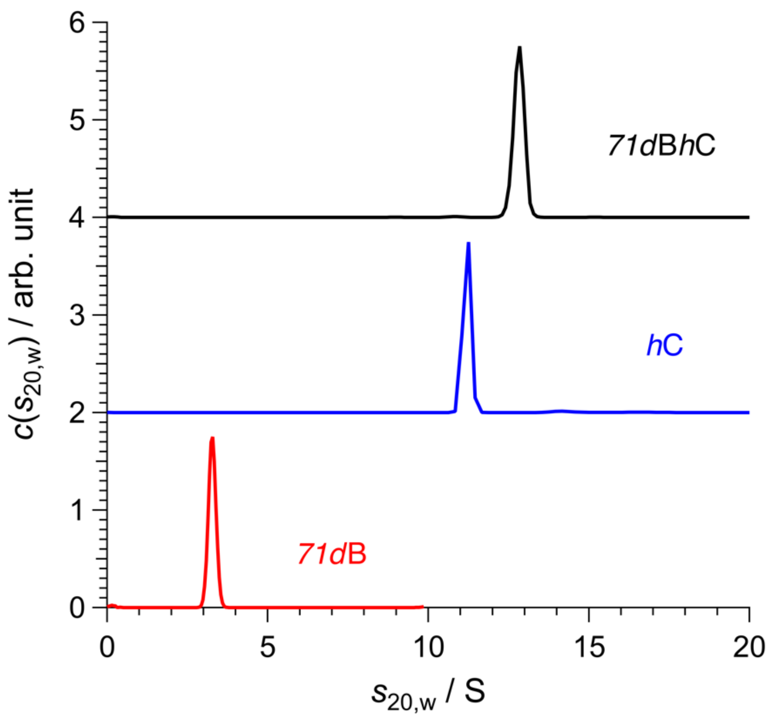


**Figure S7.** Solid black line shows the weight fraction distribution of sedimentation coefficient ($c(s_{20,w})$) for the BC complex (*71d*B*h*C) after SEC-SANS measurement. Solid red and blue lines show $c(s_{20,w})$ for free *71d*B and *h*C as references, respectively.

## S10. SAS-reporting table.

Table S4. Samples, data-collection, and analysis for OVA, BSA, and AF.

| (*a*) Sample details | | | |
|---|---|---|---|
| Organism | Gallus gallus (Chicken) | Bos taurus (Bovine) | Equus caballus (Horse) |
| Source (Catalogue No. or reference) | A7641 (Sigma) | A3641 (Sigma) | A4612 (Sigma) |
| *Scattering particle composition* | | | |
| Protein(s) | Ovalbumin (OVA) | Bovine serum albumin (BSA) | Apoferritin (AF) |
| Stoichiometry of components | Monomer | Monomer | 24-mer |
| *Sample environment/configuration* | | | |
| Solvent composition | 100 mM Tris-HCl (pH7.5), 100 mM NaCl in 99%$D_2O$ | | |
| Sample temperature (°C) | 25 | | |
| In beam sample cell | Disklike shape SEC-SANS cell with thickness of 1 mm and diameter of 18 mm (refer to Fig. S1) | | |
| *Batch measurements* | | | |
| Sample concentration(s), mg/ml | 2.1 | 2.0 | 2.5 |
| *Size Exclusion Chromatography SEC-SAS* | | | |
| Sample injection concentration, mg/ml | 7.7 | 7.9 | 12.0 |
| Sample injection volume, mL | 0.5 | 0.5 | 0.5 |
| SEC column type | Superdex 200 Increase 10/300 GL (Cytiva) | | |
| SEC flowrate, mL/min | 0.5 mL/min | | |
| (*b*) SAS data collection | | | |
| Data acquisition/reduction software | Red2D software (https://github.com/hurxl/Red2D) | | |
| Source/instrument description or reference | SANS-U at JRR-3 (Japan Atomic Energy Agency, JAEA, Ibaraki, Japan) | | |
| Measured *q*-range ($q_{min} – q_{max}$; Å$^{-1}$) | 0.01 – 0.3 | | |
| Method for scaling intensities | cm$^{-1}$ | | |
| Exposure time(s), number of exposures. | 600 sec × 24 (at SDD 4.00 m) 600 sec × 24 (at SDD 1.03 m) | 600 sec × 36 (at SDD 4.00 m) 600 sec × 24 (at SDD 1.03 m) | 600 sec × 24 (at SDD 4.00 m) 600 sec × 24 (at SDD 1.03 m) |
| Additional relevant details | $\lambda$ = 6.0 Å, $\Delta\lambda/\lambda$ = 10%, and collimator length = 4.00 m | | |
| (*c*) SAS-derived structural parameters | | | |
| | OVA | BSA | AF |
| Methods/Software | PRIMUS/qt (ATSAS 4.0.1) | | |

| | | | |
|---|---|---|---|
| *Guinier Analysis* | | | |
| $I(0) \pm \sigma$ ($cm^{-1}$) | 0.0981 ± 0.0008 | 0.136 ± 0.004 | 0.96 ± 0.01 |
| $R_g$ ± σ (Å) | 20.3 ± 0.3 | 25.6 ± 0.6 | 54.4 ± 0.8 |
| *min* < $qR_g$ < *max* limit | 0.20 < $qR_g$ < 1.30 | 0.26 < $qR_g$ < 1.30 | 0.54 < $qR_g$ < 1.30 |
| Linear fit assessment (AUTORG fidelity) | 1.00 | 1.00 | 1.00 |
| *PDDF/P(r) analysis* | | | |
| $I(0) \pm \sigma$ ($cm^{-1}$) | 0.0950 ± 0.0003 | 0.140 ± 0.002 | 0.94 ± 0.06 |
| $R_g$ ± σ (Å) | 20.0 ± 0.3 | 26.8 ± 0.3 | 54.8 ± 0.9 |
| $d_{max}$ (Å) | 60.4 | 86.5 | 231 |
| *q*-range ($Å^{-1}$) | 0.01 – 0.30 $Å^{-1}$ | 0.01 – 0.30 $Å^{-1}$ | 0.01 – 0.30 $Å^{-1}$ |
| *P*(*r*) fit assessment (CorMap P-value) | 0.871 | 0.004 | 0.089 |
| (*d*) Scattering particle size | | | |
| | OVA | BSA | AF |
| Methods/Software | PRIMUS/qt (ATSAS 4.0.1), SEDNTERP version3 | | |
| *Volume estimates* | | | |
| Porod volume, $V_p$ ($Å^3$) | 37,789 | 66,309 | 569,059 |
| *Molecular weight (M) estimates (kDa)* | | | |
| From chemical composition | 43 | 66 | 476 |
| From SAS, concentration independent method | 24 | 46 | 318 |
| From *I*(0)/concentration | 43 | 70 | 399 |
| Partial specific volume, ν ($cm^3$/g) | 0.740 | 0.735 | 0.736 |
| Contrast, $\Delta\rho$ ($cm^{-2}$) | $-3.32 \times 10^{10}$ | $-3.21 \times 10^{10}$ | $-3.17 \times 10^{10}$ |
| From SAS-independent measure (method) | n.a. | n.a. | n.a. |
| (*e*) Modelling (a complete sub-panel for each method) | | | |
| | OVA | BSA | AF |
| *Shape modelling method(s) (if used)* | n.a. | n.a. | n.a. |
| Software | | | |
| *q*-range for fit ($q_{min} - q_{max}$; $Å^{-1}$, $nm^{-1}$) | | | |
| Symmetry/anisotropy assumptions | | | |
| Number of individual model reconstructions | | | |
| $\chi^2$, CorMap *P*-values for fit | | | |
| For multiple phase models: $R_g$ values (Å, nm) | | | |

| | | | |
|---|---|---|---|
| and relative phase volumes (Å$^3$, nm$^3$) | | | |
| *Atomistic modelling methods (if used)* | | | |
| Software | CRYSON (version 2.7) | CRYSON (version 2.7) | CRYSON (version 2.7) |
| $q$-range for fit ($q_{min} – q_{max}$; Å$^{-1}$, nm$^{-1}$) | 0.01 – 0.30 Å$^{-1}$ | 0.01 – 0.30 Å$^{-1}$ | 0.01 – 0.30 Å$^{-1}$ |
| Symmetry/anisotropy assumptions | n.a. | n.a. | n.a. |
| Number of individual model reconstructions | n.a. | n.a. | n.a. |
| $\chi^2$ for fit | 1.5 | 1.3 | 2.0 |
| (*f*) Data and model deposition | | | |
| SASBDB IDs | SASDWA3 | SASDWB3 | SASDWC3 |

## Table S5. Samples, data-collection, and analysis for *h*B*h*C, and *71d*B*h*C, *h*C, and *71d*B.

| | | | | |
|---|---|---|---|---|
| (*a*) Sample details | | | | |
| Organism | *Synechococcus elongatus* PCC7942 (Cyanobacteria), | | | |
| Source (Catalogue No. or reference) | *E. coli* (BL21) recombinant expression | | | |
| *Scattering particle composition* | | | | |
| Protein(s) | Complex of Hydrogenated-KaiB and Hydrogenated-KaiC (*h*B*h*C) | Complex of 71% Deuterated-KaiB and Hydrogenated-KaiC (*71d*B*h*C) | Hydrogenated-KaiC (*h*C) | 71% Deuterated-KaiB (*71d*B) |
| Stoichiometry of components | Hexamer + Hexamer ($hB_6hC_6$) | Hexamer + Hexamer ($71dB_6hC_6$) | Hexamer ($hC_6$) | Tetramer ($71dB_4$) |
| *Sample environment/configuration* | | | | |
| Solvent composition | 50 mM sodium phosphate (pH 7.8), 150 mM sodium chloride, 5 mM magnesium chloride, 0.5 mM EDTA, 1 mM dithiothreitol, 3 mM ATP, 50 mM glutamic acid, and 50 mM arginine in 98%$D_2O$ | | | |
| Sample temperature (°C) | 25 | | | |
| In beam sample cell | Disklike shape SEC-SANS cell with thickness of 1 mm and diameter of 18 mm (refer to Fig. S1) | | | |
| *Batch measurements* | | | | |
| Sample concentration(s), mg/ml | 1.5 | 1.9 | 1.1 | 0.6 |
| *Size Exclusion Chromatography SEC-SAS* | | | | |
| Sample injection concentration, mg/ml | 6.8 | 6.8 | 5.6 | 1.2 |
| Sample injection volume, mL | 0.5 | | | |
| SEC column type | Superdex 200 Increase 10/300 GL (Cytiva) | | | |
| SEC flowrate, mL/min | 0.5 mL/min | | | |
| (*b*) SAS data collection | | | | |
| Data acquisition/reduction software | Red2D software (https://github.com/hurxl/Red2D) | | | |
| Source/instrument description or reference | SANS-U at JRR-3 (Japan Atomic Energy Agency, JAEA, Ibaraki, Japan) | | | |
| Measured *q*-range ($q_{min} – q_{max}$; $Å^{-1}$) | 0.01 – 0.3 | | | |
| Method for scaling intensities | $cm^{-1}$ | | | |
| Exposure time(s), number of exposures. | 600 sec × 12 (at SDD 4.00 m) 600 sec × 12 (at SDD 1.03 m) | 600 sec × 24 (at SDD 4.00 m) 600 sec × 24 (at SDD 1.03 m) | 600 sec × 24 (at SDD 4.00 m) 600 sec × 24 (at SDD 1.03 m) | 600 sec × 12 (at SDD 4.00 m) 600 sec × 12 (at SDD 1.03 m) |
| Additional relevant details | $\lambda$ = 6.0 Å, $\Delta\lambda/\lambda$ = 10%, and collimator length = 4.00 m | | | |
| (*c*) SAS-derived structural parameters | | | | |
| | *h*B*h*C | *71d*B*h*C | *h*C | *71d*B |
| Methods/Software | PRIMUS/qt (ATSAS 4.0.1) | | | |

| | | | | |
|---|---|---|---|---|
| *Guinier Analysis* | | | | |
| $I(0) \pm \sigma$ ($cm^{-1}$) | 0.584 ± 0.008 | 0.547 ± 0.008 | 0.393 ± 0.005 | n.a. |
| $R_g$ ± σ (Å) | 49.1 ± 0.9 | 43.2 ± 0.6 | 43.7 ± 0.7 | n.a. |
| *min* < $qR_g$ < *max* limit | 0.50 < $qR_g$ < 1.30 | 0.43 < $qR_g$ < 1.30 | 0.44 < $qR_g$ < 1.30 | n.a. |
| Linear fit assessment (AUTORG fidelity) | 0.99 | 0.99 | 0.99 | n.a. |
| *PDDF/P(r) analysis* | | | | |
| $I(0) \pm \sigma$ ($cm^{-1}$) | 0.580 ± 0.005 | 0.540 ± 0.003 | 0.389 ± 0.003 | n.a. |
| $R_g$ ± σ (Å) | 43.3 ± 0.6 | 41.2 ± 0.5 | 40.8 ± 0.4 | n.a. |
| $d_{max}$ (Å) | 122.9 | 116.4 | 119.2 | n.a. |
| *q*-range (Å$^{-1}$) | 0.01 – 0.30 Å$^{-1}$ | 0.01 – 0.30 Å$^{-1}$ | 0.01 – 0.30 Å$^{-1}$ | n.a. |
| *P*(*r*) fit assessment (CorMap P-value) | 0.028 | 0.048 | 0.862 | n.a. |

(*d*) Scattering particle size

| | *h*B*h*C | *71d*B*h*C | *h*C | *71d*B |
|---|---|---|---|---|
| Methods/Software | PRIMUS/qt (ATSAS 4.0.1), SEDNTERP version3 | | | |
| *Volume estimates* | | | | |
| Porod volume, $V_p$ (Å$^3$) | 351,403 | 287,066 | 284,265 | n.a. |
| *Molecular weight (M) estimates (kDa)* | | | | |
| From chemical composition | 427 | 427 | 356 | 47 |
| From SAS, concentration independent method | 208 | 170 | 186 | n.a. |
| From *I*(0)/concentration | 442 | 333 | 334 | n.a. |
| Partial specific volume, ν ($cm^3/g$) | 0.740 | 0.735 | 0.737 | 0.730 |
| Contrast, $\Delta\rho$ ($cm^{-2}$) | $-3.17 \times 10^{10}$ | $-2.65 \times 10^{10}$ | $-3.32 \times 10^{10}$ | $-4.62 \times 10^{13}$ |
| From SAS-independent measure (method) | n.a. | n.a. | n.a. | n.a. |

(*e*) Modelling (a complete sub-panel for each method)

| | *h*B*h*C | *71d*B*h*C | *h*C | *71d*B |
|---|---|---|---|---|
| *Shape modelling method(s) (if used)* | n.a. | n.a. | n.a. | n.a. |
| Software | | | | |
| *q*-range for fit ($q_{min} - q_{max}$; Å$^{-1}$, $nm^{-1}$) | | | | |
| Symmetry/anisotropy assumptions | | | | |
| Number of individual model reconstructions | | | | |
| $\chi^2$, CorMap *P*-values for fit | | | | |
| For multiple phase models: $R_g$ values (Å, nm) | | | | |

| | | | | |
|---|---|---|---|---|
| and relative phase volumes (Å$^3$, nm$^3$) | | | | |
| *Atomistic modelling methods (if used)* | n.a. | n.a. | n.a. | n.a. |
| Software | | | | |
| $q$-range for fit ($q_{min} – q_{max}$; Å$^{-1}$, nm$^{-1}$) | | | | |
| Symmetry/anisotropy assumptions | | | | |
| Number of individual model reconstructions | | | | |
| $\chi^2$, CorMap $P$-values for fit | | | | |
| (*f*) Data and model deposition | | | | |
| SASBDB IDs | SASDWD3 | SASDWE3 | SASDWF3 | n.a. |
